# Multinodular Vacuolating Neuronal Tumors: Symptomatic Presentation Versus Incidental Finding: Case Series and Literature Review

**DOI:** 10.3390/reports7040086

**Published:** 2024-10-23

**Authors:** Arturs Balodis, Sintija Strautmane, Paula Mežvinska, Sergejs Pavlovičs

**Affiliations:** 1 Department of Radiology, Riga Stradins University, LV-1007 Riga, Latvia; 2 Institute of Diagnostic Radiology, Pauls Stradins Clinical University Hospital, LV-1002 Riga, Latvia; 3 Faculty of Residency, Riga Stradins University, LV-1007 Riga, Latvia; 4 Department of Neurology, Pauls Stradins Clinical University Hospital, LV-1002 Riga, Latvia; 5 Faculty of Medicine, Riga Stradins University, LV-1007 Riga, Latvia; 040298@rsu.edu.lv

**Keywords:** multinodular vacuolating neuronal tumor (MVNT), epilepsy, supratentorial tumor, subcortical and cortical nodules

## Abstract

**Background and Clinical Significance:** Multinodular Vacuolating Neuronal Tumors (MVNTs) are mixed glial–neuronal brain lesions classified as World Health Organization (WHO) CNS grade 1 tumors, often associated with long-term epilepsy. First described by Huse et al. in 2013 and included in the WHO CNS classification in 2016, MVNTs present a range of clinical manifestations, from symptomatic to asymptomatic. They typically affect young to middle-aged adults and exhibit diverse presentations. Radiologically, MVNTs are usually supratentorial, frequently located in the temporal lobe but also observed in the frontal and parietal lobes. MRI is essential for diagnosis, revealing multiple coalescing subcortical or cortical nodules with hyperintense signals on T2-weighted/FLAIR sequences, often without peripheral edema or mass effects. **Case Presentation:** This paper presents two cases: one symptomatic MVNT with significant clinical manifestations, and the other documenting an incidental finding of MVNT in an asymptomatic patient. One case shows typical temporal lobe localization, while the other highlights a rare frontal lobe localization, with clear radiological findings on T2/FLAIR sequences. **Conclusions:** These cases illustrate the varied clinical presentations of MVNTs and emphasize MRI’s critical role in diagnosis and management. Asymptomatic cases often require conservative management, stressing the avoidance of unnecessary invasive procedures and the importance of regular monitoring.

## 1. Introduction and Clinical Significance

Multinodular vacuolizing neuronal tumors (MVNTs) are benign (WHO CNS Grade 1), mixed glial–neuronal brain lesions classified under long-term epilepsy-associated tumors (LEATs). First described by Huse et al. in 2013 through the analysis of ten patient cases, these tumors were only incorporated into the World Health Organization (WHO) CNS tumor classification in 2016. According to the updated 2021 WHO CNS tumor classification, MVNTs are categorized among glioneuronal and neuronal tumors [1,2,3]. In some cases of MVNTs, molecular alterations in the RAS/MAPK pathway have been identified, particularly mutations in exon 2 of MAP2K1. However, genetic analyses have not demonstrated consistent changes, indicating a need for further research to elucidate the molecular characteristics and pathogenesis of these tumors [2,4].

MVNTs are typically diagnosed in young to middle-aged adults, with an average age ranging from 38 to 44 years [5,6]. Rarely, MVNTs also affect the pediatric population [7,8]. Although several case series have reported a higher prevalence in females, precise epidemiological data remain scarce, largely due to the relatively recent discovery of MVNTs and their often asymptomatic courses [1,5,9].

This paper presents two case reports of MVNT. The first case features a symptomatic MVNT with significant clinical manifestations and an unusual frontal lobe localization. The second case describes an incidental MVNT discovered in an asymptomatic patient, with a typical temporal lobe localization. Both cases are characterized by distinctive radiological findings on T2/FLAIR sequences.

## 2. Case Presentation

### 2.1. Case 1

A 59-year-old woman was admitted to the neurology department for an extended 48 h electroencephalography (EEG). An outpatient brain magnetic resonance imaging (MRI) had previously revealed a lesion in the right frontal lobe. For approximately three months, the patient had been experiencing severe, pressing headaches, which were alleviated with gabapentin, 100 mg, twice daily. Additionally, she reported memory impairments, difficulty making decisions, balance issues, particularly when looking to the left, disturbances in taste and smell, visual perception issues, and blurred vision. She had developed slurred speech for three weeks

She also complained of dropping objects from her left hand, water dribbling from her mouth while drinking, and pain and sensory disturbances on the left side of her face. The patient denied any weight loss or bleeding, and she has multiple drug allergies. She had been diagnosed with grade 2 primary arterial hypertension and dyslipidemia.

During her hospitalization, the patient noted a numbness sensation on the plantar surface of her left foot, which had been present for more than six months. These symptoms occurred in the morning after getting out of bed, interfered with movement, and resolved within 15 to 90 min. No other complaints were noted during the examination. Neurological examination revealed bilaterally weakened tendon reflexes in the arms and legs, hypoesthesia to pain stimuli on the lateral surface of the left forearm, the plantar surface of the left foot, and the anterior surface of the left lower leg.

**Figure 1:**
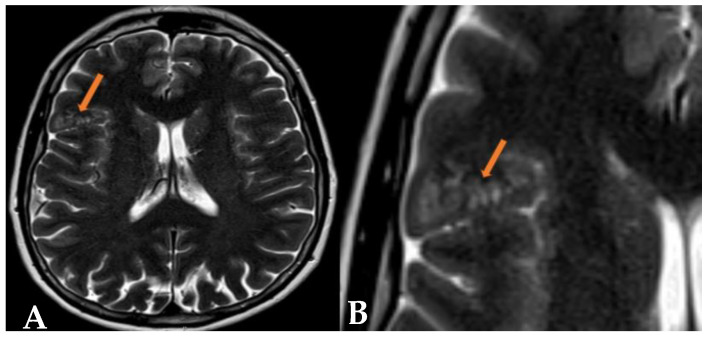
(**A**,**B**) In the T2-weighted axial images, there are hyperintense vacuolating round nodules predominantly located in the subcortical region of the right frontal lobe. These nodules, which resemble “bubbles”, appear to slightly extend into the cortical area (orange arrow). When correlated with findings from other MRI sequences, these features are highly suggestive of Multinodular and Vacuolating Neuronal Tumors (MVNT).

**Figure 2:**
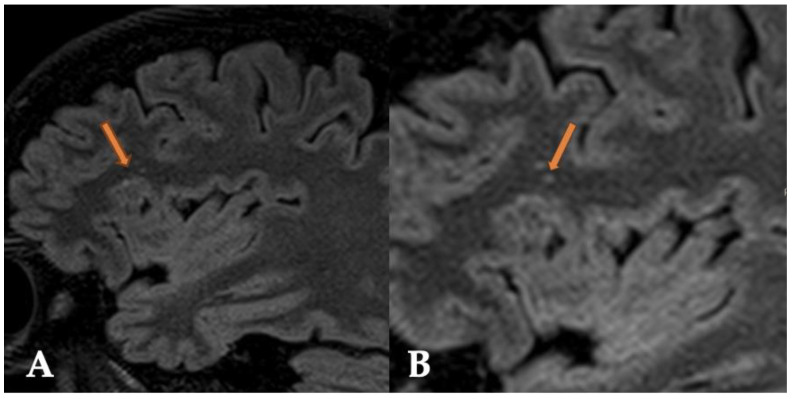
The FLAIR sequence in the sagittal plane (**A**,**B**) shows hyperintense, vacuolating, round nodules predominantly localized subcortically, with some likely involving the cortex as well (orange arrow).

**Figure 3:**
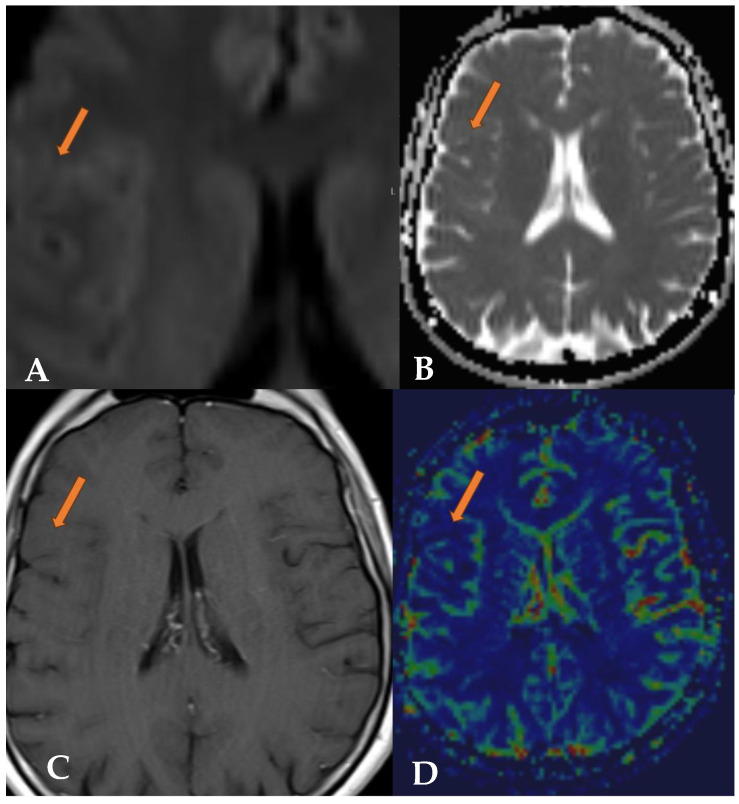
MRI DWI (**A**) and ADC maps (**B**), as well as T1 images after Gadovist contrast administration (**C**) and MRI perfusion images (**D**), are visible. In these MRI series, no diffusion restriction is seen, there is no decrease in ADC values, and no contrast enhancement is observed (orange arrow). The CBV in perfusion is low, indicating no signs of potential malignancy.

A repeat MRI with intravenous contrast showed T2 and FLAIR hyperintense multinodular cystic leasions in the right frontal lobe, primarily cortical and subcortical along the inferior frontal gyrus (Figure 1 and Figure 2). The lesion did not exhibit contrast enhancement, and there were no signs of increased diffusion signal or apparent diffusion coefficient (ADC) value reduction (Figure 3). No significant changes in the lesion were observed compared to previous examinations. The radiological findings were consistent with a multinodular vacuolating neuronal tumor. Based on these findings, a neurosurgeon was consulted, who recommended a follow-up MRI in six months with subsequent outpatient neurosurgical consultation. Currently, there are no indications for neurosurgical intervention.

The 48 h EEG conducted during hospitalization did not register any pathological slowing or epileptiform activity. Blood tests showed the presence of CASPR2 IgG antibodies, leading to plans for a lumbar puncture, as well as computed tomography (CT) scans of the lungs and abdomen with intravenous contrast. However, the patient declined these examinations. Given the patient’s stable condition, she was discharged for further outpatient monitoring and treatment.

### 2.2. Case 2

A 48-year-old female patient was admitted to the neurology department with complaints of pain in the thoracic and lumbar spine, radiating to the left leg down to the knee, and spreading along the back of the thigh, as well as pain in both shoulder joints. At the time of examination, the pain intensity was assessed as a 6 on the Numeric Assessment Scale (NAS). Neurological examination revealed mild pronation of the right hand, signs of ataxia in both legs during heel-knee tests, and a positive Babinski reflex on the right side. Palpation revealed pain in the thoracic and lumbar spine, as well as in the right hip joint area, confirmed by a bilaterally positive FABER test. Hypoesthesia was observed in the right hand, and polyneuritic-type sensory disturbances were noted in the legs.

**Figure 4:**
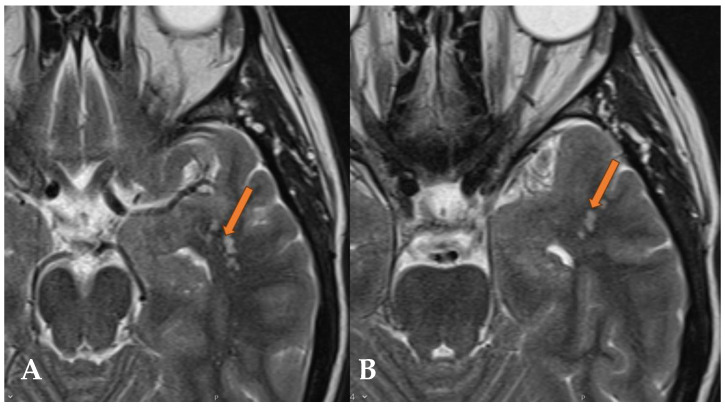
(**A**,**B**) In the T2 axial images, predominantly subcortical in the anterior parts of the superior and middle gyri of the left temporal lobe, hyperintense vacuolating round nodules resembling ‘bubbles’ are seen (orange arrow). Considering the changes in other MRI series, the finding is more likely characteristic of Multinodular and Vacuolating Neuronal Tumors (MVNT).

**Figure 5:**
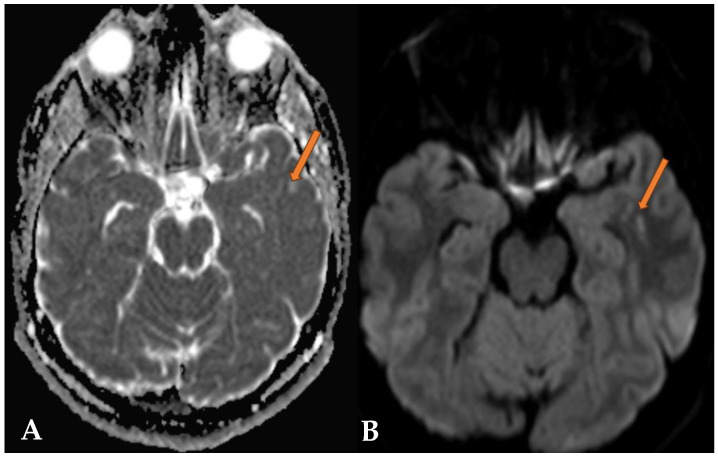
The MRI DWI (**B**) and ADC map (**A**) show no diffusion restriction, and the ADC map value is high (orange arrow).

**Figure 6:**
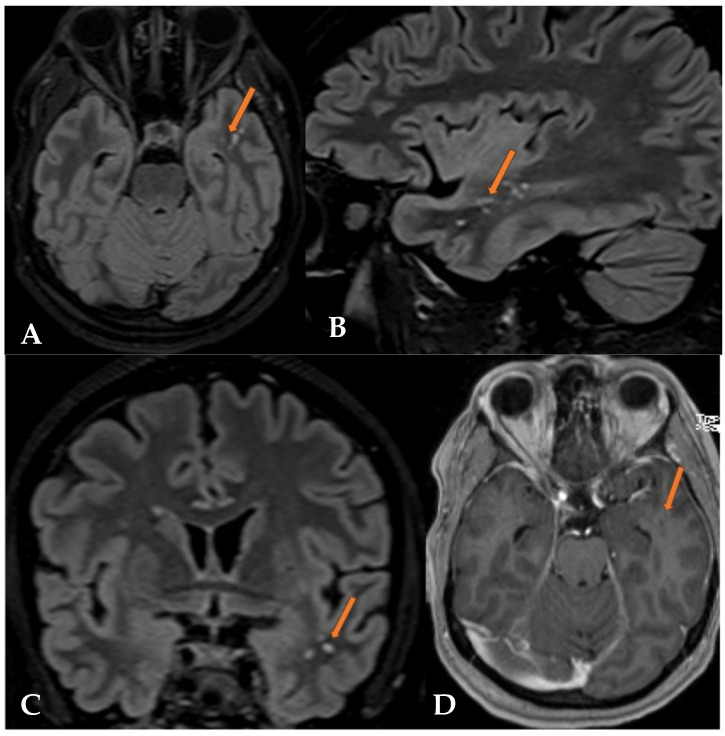
The FLAIR sequence in the axial (**A**), sagittal (**B**), and coronal (**C**) planes shows hyperintense vacuolating round nodules, predominantly localized subcortically, but also likely slightly cortically in the left temporal lobe (orange arrow). T1 after contrast in the axial plane (**D**) shows no enhancement in the visible nodular areas of the left temporal lobe.

Anamnesis data indicate that, four years ago, the patient suffered a lacunar cerebral infarction in the territory of the left posterior cerebral artery (PCA). Following this event, repeated brain magnetic resonance imagings (MRIs) and magnetic resonance angiographies (MRAs) were performed to assess dynamic changes. The investigations identified not only old lacunar ischemia in the left thalamus within the PCA territory, but also focal changes in the left hemisphere, particularly in the middle and superior gyri of the temporal lobe (Figure 4, Figure 5 and Figure 6). These findings corresponded with the radiological features of a Multinodular and Vacuolating Neuronal Tumor (MVNT). Compared to previous MRI scans, no dynamic changes were observed. Given that the patient had no complaints related to MVNT, this finding is considered incidental.

During hospitalization, the patient underwent comprehensive diagnostic examinations and received intravenous analgesic therapy. Doppler ultrasonography revealed 10–20% stenosis of the proximal left internal carotid artery, while a computed tomography (CT) scan showed lumbar spine spondylosis, spondylarthrosis, L5-S1 disk protrusions with left L5-S1 radicular pain syndrome, cervical spine spondylosis, spondylarthrosis, foraminal stenosis with bilateral C5-C6 radicular pain syndrome, and right hip joint peritrochanteritis. Additionally, electroneurography diagnosed bilateral carpal tunnel syndrome. The patient also has a history of coronary artery disease, grade 2 primary arterial hypertension, dyslipidemia, and gastric ulcers.

A repeat brain MRI showed no dynamic changes in the lesion compared to previous examinations. It is recommended that the patient undergo a follow-up brain MRI with intravenous contrast in one year to ensure that the lesion does not progress. Currently, a biopsy of the lesion is not necessary, as it is asymptomatic and shows no signs of progression.

## 3. Discussion

The two cases presented in this paper align with the broader literature on MVNTs, which highlights the diverse clinical and radiological characteristics of these lesions. Typically, these lesions are located supratentorially, but radiologically similar lesions have also been described infratentorially, referred to as multinodular and vacuolizing posterior fossa lesions (MV-PLUS), though these have not yet been histologically confirmed [10,11,12]. Shitara et al. found that MVNTs predominantly affect the temporal lobe, accounting for 64.7% of cases. [13]. However, larger case series also report significant involvement of the frontal and parietal lobes. Nunes et al. noted that among 33 patients, 9 (27%) had tumors in the parietal lobe, 8 (24%) in the frontal lobe, and 6 (18%) in the temporal lobe. Lecler et al. found that MVNTs were predominantly localized to the parietal (26/64) and frontal (20/64) lobes [1,6]. These tumors frequently localize in the temporal lobe, which may be related to the fact that this region often causes seizures rather than an inherent predisposition for tumor development. MVNTs often localize in the subcortical white matter [9], hippocampus [7,14,15], amygdala [2,14,15], as well as the basal ganglia [7,16], thalamus, fornix, septum pellucidum, and corpus callosum [16].

Clinically, MVNTs present with a diverse spectrum of symptoms including epilepsy, headaches, and other nonspecific neurological complaints. However, MVNTs frequently exhibit an asymptomatic course, and tumors are often diagnosed incidentally [13,17,18,19]. MVNTs are commonly associated with epilepsy that is resistant to pharmacological control, and improvement or resolution of symptoms often occurs only after tumor resection [8,14,20]. Baščarevič et al. found that approximately 41% of 96 patients suspected of MVNT also had epilepsy [21]. Headaches are another common complaint among MVNT patients. Nunes et al. reported headaches in 16/33 patients [1]. Biyikli et al. also found headaches to be the most frequent complaint, affecting 7/11 patients [5]. One of our patients also experienced persistent headaches, illustrating the importance of considering MVNTs in the differential diagnosis of chronic and unexplained headaches. Nonetheless, symptoms are not always directly related to the tumor’s location [22]. Do et al. described a case where MVNT presented with sudden, severe headaches (thunderclap headaches), initially misdiagnosed as acute cerebral infarction [23]. In rare cases, other neurological symptoms such as dizziness [2,15,24], attention disturbances, dysarthria, blurred vision, episodic numbness in the arms, legs, or back, tingling vertigo, confusion [2], postural tone loss, cognitive and language disturbances, paresthesia, hypoesthesia [9,25,26], and stroke-like symptoms have been observed [5]. However, Nunes et al. reported that only 4 out of 33 patients had clinical symptoms potentially related to the tumor [1]. Sirbu et al. described a case where seizures in a patient were possibly triggered by alcohol or drug use, highlighting external factors that might cause symptoms in otherwise asymptomatic tumors [27].

In diagnosing MVNTs, brain MRI is crucial, as it provides more informative data compared to computed tomography (CT) scans. In several cases, CT may reveal no abnormalities or only subcortical hypoattenuation without calcification or cortical abnormalities [14,27,28,29]. Biyikli et al. found that tumors were not identified in CT scans for five of six patients (83%) [5]. Both of our cases demonstrated the characteristic MRI features of MVNTs, including multiple coalescing subcortical or cortical nodules that were hyperintense on T2-weighted images (T2WI) and fluid-attenuated inversion recovery (FLAIR) sequences, iso- to hypointense on T1-weighted images (T1WI) compared to gray matter, and with no associated mass effects or edema, though a minor mass effect has been observed in some cases [30]. They do not show restricted diffusion on diffusion-weighted imaging (DWI) or blooming on gradient echo (GRE)/susceptibility-weighted imaging (SWI) [9]. In most cases, these tumors do not exhibit contrast enhancement after gadolinium administration, differentiating them from other neoplastic lesions. However, some cases have demonstrated contrast enhancement [1,2,17]. Alsufayan et al. reported contrast enhancement in three lesions (13%), showing a punctate and linear pattern [9].

Radiologically, MVNTs bear some resemblance to other LEATs, such as dysembryoplastic neuroepithelial tumors (DNETs), low-grade gliomas (e.g., gangliogliomas), and expanded Virchow–Robin spaces (EPVS). Thus, these conditions should be considered in the differential diagnosis of MVNTs [25,30,31]. DNETs, like MVNTs, are associated with epilepsy, often involve the temporal lobe, and can be located subcortically or cortically, with MRI findings similar to MVNTs—hyperintense on T2WI and FLAIR without mass effect or edema [32]. Due to these similarities, MVNTs have been misinterpreted as DNETs in several cases [2,14,20]. Pak et al. concluded that MRI features such as a diffusion restriction sign and the absence of cortical involvement are reliable radiological markers for differentiating these two pathologies. Cortical involvement was present in only 10% of MVNT cases, compared to 100% in DNET cases. The “bubble-like” appearance had high sensitivity (~94%) but low specificity (~36%) [33]. Additionally, FLAIR sequences can help differentiate EPVS from MVNTs, as EPVS appears hypointense while MVNTs appear hyperintense [34]. Due to the similar radiological appearance, MVNTs are sometimes confused with low-grade gliomas. Huse et al. and Gonzalez-Quarante et al. reported that some cases were treated with radiotherapy due to initial suspicion of low-grade gliomas [2,30]. Choi et al. performed a retrospective study where initial diagnoses of gangliogliomas, gangliocytomas, and focal cortical dysplasia were later revised to MVNTs based on typical histological features of MVNTs [17].

In the literature, MVNTs are consistently described as “leave me alone” tumors due to their benign nature. Nunes et al. found that most tumors remained stable during a follow-up period of 24 months to 3 years, with biopsies performed in only four cases due to symptomatic manifestations, supporting a conservative approach for asymptomatic MVNTs [1]. Neurosurgical intervention is employed when a patient’s epileptic seizures or other symptoms are clearly linked to the MVNT. In clinical cases reported in the literature with available follow-up data, most patients experienced symptom resolution or improvement after partial or complete tumor resection. Bodi et al. described a case where postoperative seizure frequency decreased by 50%, although seizures did not completely resolve [20]. Overall, the prognosis for these tumors is considered favorable, as tumor recurrence is rarely reported even with partial resection. An exception was a case in Alsufayan et al.’s series where 1/24 patients showed bilateral frontal lobe tumor expansion on follow-up MRI after 3 months, and this tumor was one of the few in their series that exhibited contrast enhancement [9].

In 2024, De Simone M et al. published an article about advancements in glioma care, focusing on emerging neurosurgical techniques according to the new classification system of gliomas [35]. However, further developments in the field of neurosurgery are warranted to provide the best possible outcome for the patient.

One of the neurosurgical methods gaining popularity is the transorbital approach, for various brain lesions. A detailed article on this topic was published in 2024, written by De Simone M et al., aiming to enhance the awareness and knowledge regarding the current utility of the transorbital approach in neurosurgery [36]. Surgical management of MVNT gives a pathologic diagnosis of the tumor; however, in asymptomatic patients, careful monitoring may be sufficient.

The diagnosis of MVNT in our cases was based exclusively on MRI findings, a decision driven by the tumor’s highly characteristic imaging appearance. MVNTs present as multiple coalescing nodules that are hyperintense on T2WI and FLAIR sequences, typically without mass effects, edema, or contrast enhancement. These distinct features, extensively documented in the literature, establish MRI as a reliable diagnostic tool. Similar published clinical cases also rely on radiological diagnosis without histological confirmation. Given the benign and stable nature of MVNTs, the risk associated with an invasive biopsy was deemed unnecessary, particularly in asymptomatic cases. Instead, a conservative approach with regular MRI monitoring was chosen, ensuring patient safety while allowing for timely intervention if needed. This approach is further validated by studies showing that most MVNTs remain stable over time, with very few cases exhibiting any signs of progression.

## 4. Conclusions

In summary, these cases illustrate the diverse clinical presentations of MVNTs and emphasize the critical role of MRI in their diagnosis and management. The variability in symptoms underscores the necessity for thorough neurological and radiological evaluation to ensure accurate diagnosis and tailored treatment. The conservative management approach adopted aligns with current best practices, highlighting the importance of personalized care based on tumor characteristics and patient condition. To enhance understanding of MVNTs, further research with larger case series is needed to explore their long-term behavior and potential for growth or malignant transformation.

## Data Availability

The data presented in this study are available on request from the corresponding author. The data are not publicly available due to privacy restrictions.
